# Gene expression changes in sickle cell reticulocytes and their clinical associations

**DOI:** 10.1038/s41598-023-40039-2

**Published:** 2023-08-08

**Authors:** Xu Zhang, Jihyun Song, Binal N. Shah, Jin Han, Taif Hassan, Galina Miasniakova, Adelina Sergueeva, Sergei Nekhai, Roberto F. Machado, Mark T. Gladwin, Santosh L. Saraf, Josef T. Prchal, Victor R. Gordeuk

**Affiliations:** 1 Department of Medicine, University of Illinois at Chicago, Chicago, IL USA; 2 Department of Medicine, University of Utah, Salt Lake City, UT USA; 3 College of Pharmacy, University of Illinois at Chicago, Chicago, IL USA; 4 Chuvash Republic Clinical Hospital 2, Cheboksary, Russia; 5 Cheboksary Children’s Hospital, Cheboksary, Russia; 6 Center for Sickle Cell Disease, Howard University, Washington, DC USA; 7 Division of Pulmonary, Critical Care, Sleep, and Occupational Medicine, Department of Medicine, Indiana University, Indianapolis, IN USA; 8 Division of Pulmonary, Allergy, and Critical Care Medicine, Vascular Medicine Institute, University of Pittsburgh, Pittsburgh, PA USA

**Keywords:** Genetics research, Translational research

## Abstract

Transcriptional changes in compensatory erythropoiesis in sickle cell anemia (SCA) and their disease modulation are unclear. We detected 1226 differentially expressed genes in hemoglobin SS reticulocytes compared to non-anemic hemoglobin AA controls. Assessing developmental expression changes in hemoglobin AA erythroblasts for these genes suggests heightened terminal differentiation in early erythroblasts in SCA that diminishes toward the polychromatic to orthochromatic stage transition. Comparison of reticulocyte gene expression changes in SCA with that in Chuvash erythrocytosis, a non-anemic disorder of increased erythropoiesis due to constitutive activation of hypoxia inducible factors, identified 453 SCA-specific changes attributable to compensatory erythropoiesis. Peripheral blood mononuclear cells (PBMCs) in SCA contain elevated proportions of erythroid progenitors due to heightened erythropoiesis. Deconvolution analysis in PBMCs from 131 SCA patients detected 54 genes whose erythroid expression correlated with erythropoiesis efficiency, which were enriched with SCA-specific changes (OR = 2.9, P = 0.00063) and annotation keyword “ubiquitin-dependent protein catabolic process”, “protein ubiquitination”, and “protein polyubiquitination” (OR = 4.2, P = 7.5 × 10^–5^). An erythroid expression quantitative trait locus of one of these genes, *LNX2* encoding an E3 ubiquitin ligase, associated with severe pain episodes in 774 SCA patients (OR = 1.7, P = 3.9 × 10^–5^). Thus, erythroid gene transcription responds to unique conditions within SCA erythroblasts and these changes potentially correspond to vaso-occlusive manifestations.

## Introduction

In sickle cell anemia (SCA), polymerization of deoxygenated hemoglobin S results in erythrocytes with reduced deformability^1^, altered properties of flow and aggregation^2^, and high membrane conductance of cations^3^. Abnormal display of adhesive surface molecules induces erythrocyte interaction with endothelial cells and other blood cells to occlude microvasculature^4^. Damaged erythrocytes rupture or are removed from circulation by phagocytes at a much younger age of 17–35 days than 100–120 days in normal individuals^5^. Compensatory erythropoiesis occurs in response to hemolytic anemia and tissue hypoxia in SCA^6^. Reticulocytes in SCA constitute 4–15% or more of total erythrocytes compared to 0.5–1.5% in normal individuals.

Polymerization of hemoglobin S also occurs in bone marrow erythroblasts that synthesize and accumulate hemoglobin^7^, thereby impacting erythropoiesis^8^. High cell death rates occur between the polychromatic and orthochromatic maturation stages of in vitro and in vivo derived SCA erythroblasts^9^. The phenomenon is reminiscent of ineffective erythropoiesis in β-thalassemia major and intermedia, characterized by accelerated erythroid differentiation, maturation arrest, and apoptosis at the polychromatic stage^10^, although ineffective erythropoiesis is less marked in SCA versus β-thalassemia.

The pattern and regulation of transcription changes characterizing compensatory erythropoiesis in SCA are largely unknown. In derived human erythroblasts, global transcriptional activation peaks in proerythroblasts and reduces to 40% of the peak in polychromatic and orthochromatic stages^11^. Distinct sets of genes are activated at stage transitions throughout erythroid differentiation^12,13^. Reticulocytes retain transcripts from erythroblasts^14^; therefore differential gene expression observed in reticulocytes between SCA patients and healthy individuals can reflect cumulative variation in the preceding stages. Importantly, some observations suggest contribution of reticulocytes to vaso-occlusion manifested as acute painful episodes^4,15,16^. We postulated that reticulocyte transcriptional variation may give rise to certain disease manifestations.

We applied RNA-Seq to reveal gene expression changes in reticulocytes from SCA patients compared to healthy African Americans. We delineated SCA-specific regulation of reticulocyte transcripts using gene expression changes in Chuvash erythrocytosis (CE) as a comparator^17^. CE is a monogenic disorder characterized by homozygosity for hypomorphic *VHL*^R200W^ leading to constitutive hypoxia inducible factor (HIF) activation that results in increased erythropoietin, erythrocytosis and a propensity to thrombosis^18^. We assessed clinical correlations with erythroid expression variation employing deconvolution analysis^19^ of archived gene expression data from SCA peripheral blood mononuclear cells (PBMCs), which contain elevated erythroid progenitors due to heightened erythropoiesis^20^. We further assessed genetic association between erythroid expression quantitative trait loci (eQTL) and clinical manifestations.

## Results

**Figure 1:**
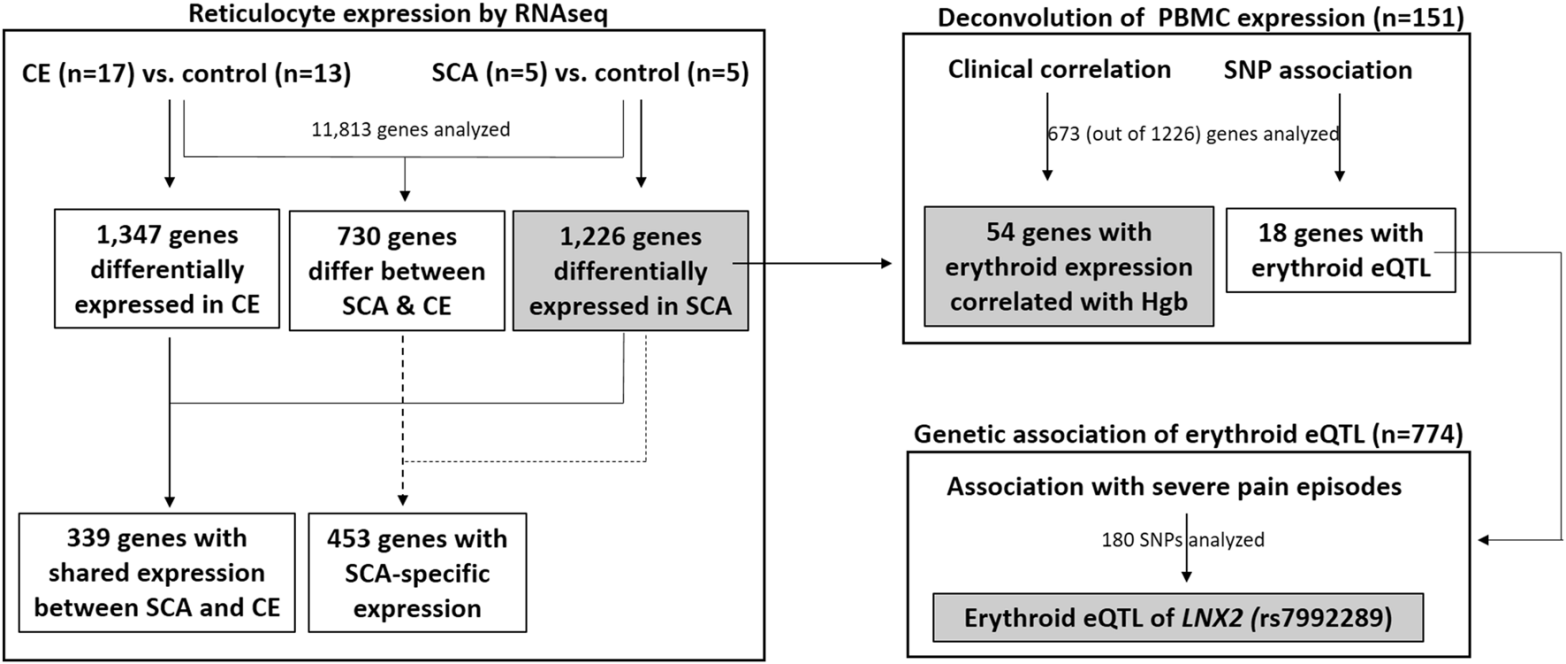
Flow chart of data analyses.

A flow chart of data analyses is presented in Fig. 1.

### Gene expression changes in SCA reticulocytes

**Figure 2:**
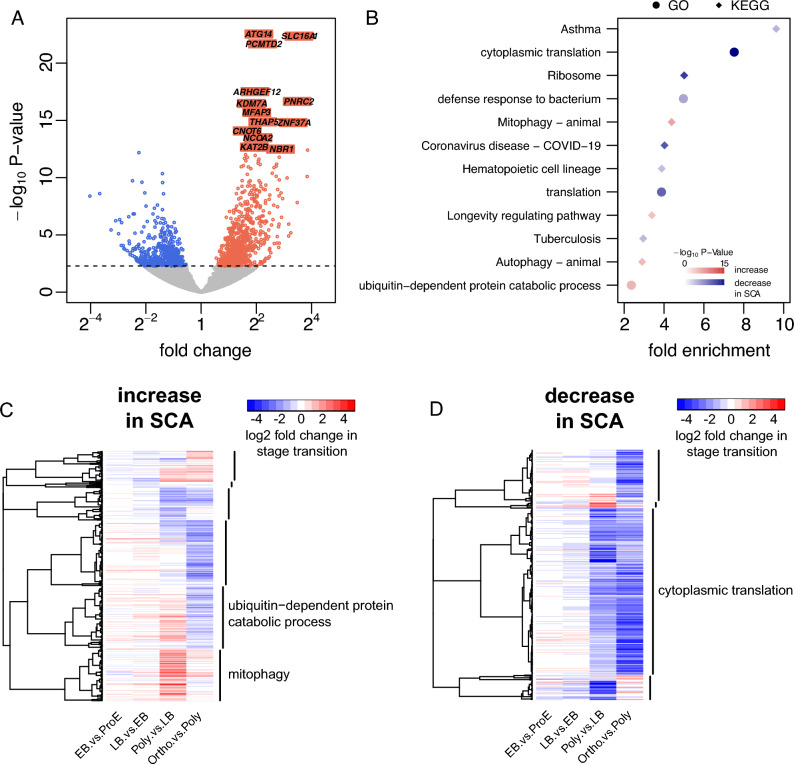
Differential genes detected at 5% FDR in hemoglobin SS reticulocytes from 5 SCA patients compared to hemoglobin AA reticulocytes from 5 African American control individuals. (**A**) Volcano plot of differential genes. The top one percent of the most signifcant genes were labeled. *ATG14* autophagy related 14, *SLC16A1* solute carrier family 16 member 1, *PCMTD2* protein-L-isoaspartate (D-aspartate) O-methyltransferase domain containing 2, *ARHGEF12* Rho guanine nucleotide exchange factor 12, *PNRC2* proline rich nuclear receptor coactivator 2, *KDM7A* lysine demethylase 7A, *MFAP3* microfibrillar associated protein 3, *THAP5* THAP domain containing 5, *ZNF37A* zinc finger protein 37A, *CNOT6* CCR4-NOT transcription complex subunit 6, *NCOA2* nuclear receptor coactivator 2, *KAT2B* lysine acetyltransferase 2B, *NBR1* NBR1 autophagy cargo receptor. (**B**) GO biological processes and KEGG pathways enriched with differential genes. (**C,D**) Hierarchical clustering of genes whose expressions were (**C**) increased or (**D**) decreased in hemoglobin SS reticulocytes, using correlation distance of expression changes across stage transitions in hemoglobin AA erythrobasts by An et al. Enriched GO biological processes with adjusted P-value < 0.05 are labelled per gene cluster.

Five SCA patients (mean ± standard deviation [SD] age 43 ± 8 years; 80% female; mean ± SD hemoglobin concentration 73 ± 13 g/L) and 5 African-American control individuals without *HBB* mutation (mean ± SD age 44 ± 8 years; 100% female; mean ± SD hemoglobin concentration 130 ± 7.8 g/L) from the University of Illinois at Chicago (UIC) were studied. All were under steady state. SCA patients received neither hydroxyurea treatment nor blood transfusion in the three months prior to participating in the study. Principal components analysis revealed *HBB* genotype as the primary segregating variable of the studied transcriptomes (SFig. 1). At 5% false discovery rate (FDR), 1226 of 11,813 analyzed genes (10.4%) were differentially expressed in SCA compared to controls, 741 with increased expression (STable 1) and 485 with decreased expression (STable 2) in SCA. The top one percent most significant differential expressions all showed increase in SCA (Fig. 2A). Genes with increased expression in SCA are enriched in Gene Ontology (GO) biological process “ubiquitin-dependent protein catabolic process” and Kyoto Encyclopedia of Genes and Genomes (KEGG) pathway “mitophagy—animal”, “autophagy—animal”, and “longevity regulating pathway”. Genes with decreased expression in SCA are enriched in GO “cytoplasmic translation”, “translation”, and “defense response to bacterium”, as well as KEGG "ribosome", "coronavirus disease—COVID-19", "asthma", "tuberculosis", and "hematopoietic cell lineage" (STable 3, Fig. 2B). Upregulation of “mitophagy” and downregulation of “cytoplasmic translation” are consistent with clearance of mitochondria and ribosomes in terminal differentiation^21^.

Transcripts in reticulocytes are remnants of preceding erythroblasts. To assess the developmental context of the genes differentially expressed in SCA (hemoglobin SS) reticulocytes, we examined how their expressions changed in stage transitions in human hemoglobin AA erythroblasts as reported by An and colleagues^13^. An’s study applied fluorescence-activated sorting-based methods to purify erythroblasts and examined transitions spanning the proerythroblastic (ProE), early basophilic (EB), late basophilic (LB), polychromatic (Poly), and orthochromatic (Ortho) stages. Across the genes analyzed in both studies, the median reads per kilobase million in An et al. were correlated with the median fragments per kilobase million in this study with Spearman’s rho 0.40, 0.44, 0.50, 0.65, 0.68 for ProE, EB, LB, Poly, and Ortho, respectively. The increasing correlation of gene expression profile between reticulocytes and differentiating erythroblasts is consistent with the position of reticulocytes in erythroblast lineage. It also indicates that our fractioning method of reticulocyte transcripts (“Methods”) captured relevant variation in erythroblasts.

Of the 1226 differentially expressed genes in hemoglobin SS reticulocytes, 910 exhibited expression change at 5% FDR from an earlier stage to a consecutive later stage in hemoglobin AA erythroblasts in An et al.^13^ (STable 4). The direction of gene expression change in hemoglobin SS reticulocytes vs hemoglobin AA controls was consistent with the direction of expression change in the maturation of hemoglobin AA erythroblasts for decreasing proportions of genes: 82%, 83%, 70%, and 52% for stage transitions from ProE through Ortho (STable 4, test for trend in proportions P-value = 2.3 × 10^–16^). We assumed here that upregulation in SCA relative to controls of genes normally upregulated in an erythroid stage transition, or vice versa, indicates accentuated erythroid differentiation in SCA. The observed trend therefore suggests hightened erythroid differentiation in SCA relative to controls in ProE, which gradually diminishes toward Ortho. This dinimishing erythroid differentiation appeared to be driven by genes upregulated in SCA. Figure 2C shows clustering according to stage transitions of genes whose expressions were increased in SCA. Of these genes, 338 (56%) also showed increased expression from LB to Poly while only 167 (28%) had increased expression from Poly to Ortho. As shown in Fig. 2C, genes whose expressions were increased consistently from LB through Ortho are enriched in GO “mitophagy”, whereas genes whose expressions were increased from LB to Poly but then decreased from Poly to Ortho are enriched in GO “ubiquitin-dependent protein catabolic process”. Figure 2D shows clustering according to stage transitions of genes whose expressions were decreased in SCA. Of these genes, 161 (52%) also showed decreased expression from LB to Poly and 213 (69%) from Poly to Ortho. Genes whose expressions were decreased consistently from LB through Ortho are enriched in GO “cytoplasmic translation” (Fig. 2D).

### SCA-specific gene expression changes versus expression changes shared with CE

**Figure 3:**
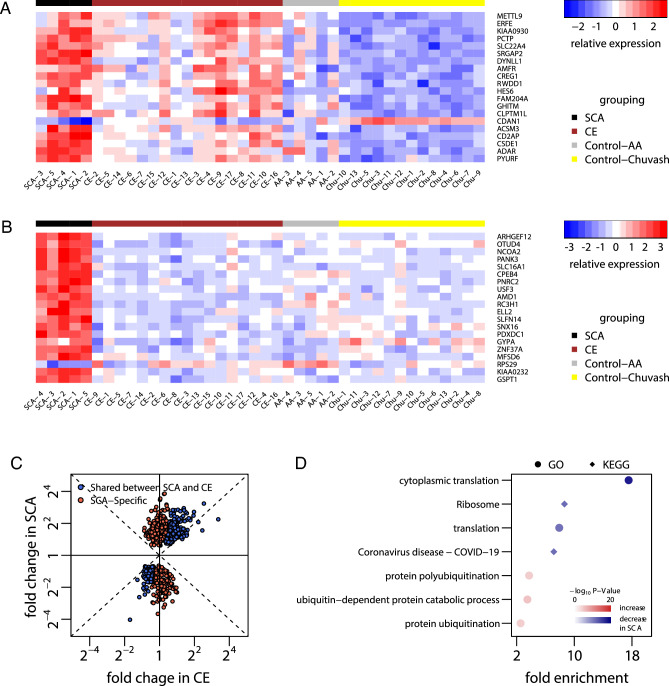
SCA-specific expression changes. (**A,B**) Hierarchical clustering of individuals within groups, using Euclidean distance of gene expression levels of (**A**) the top 20 most significant differential genes in CE whose expression changes were shared in SCA and (**B**) the top 20 most significant genes whose expression changes differed between SCA and CE. Individuals are grouped as SCA, CE, African American controls (Control-AA), and Chuvash controls (Control-Chuvash). (**C**) Scatter plot of fold change in CE (x-axis) and SCA (y-axis). (**D**) GO biological processes and KEGG pathways enriched with SCA-specific differential genes.

Heightened erythropoiesis occurs in both SCA and CE, the latter a condition of constitutive HIF activation in the absence of anemia. A comparison of gene expression changes in SCA (versus healthy African-American individuals) to gene expression changes in CE (versus healthy Chuvash individuals) allowed us to classify SCA-specific changes attributable to compensatory erythropoiesis versus changes not necessarily induced by anemia. Seventeen *VHL*^R200W^ homozygotes (mean ± SD age 48 ± 14 years; 71% female; mean ± SD hemoglobin concentration 190 ± 33 g/L) and 13 *VHL* wild-type individuals (mean ± SD age 47 ± 15 years; 77% female; mean ± SD hemoglobin concentration 129 ± 9 g/L) from Chuvashia, Russia were studied. We analyzed in CE reticulocytes the expression of the same 11,813 genes as in SCA reticulocytes. Of the 1347 differentially expressed genes detected in CE reticulocytes at 5% FDR, 339 shared the same direction of expression change with SCA reticulocytes, 220 having increased and 119 decreased expression (STables 1 and 2). Figure 3A shows the top 20 most significant differential genes in CE whose expression changes were shared in SCA.

We performed a formal genome-wide comparison between SCA and CE of reticulocyte gene expression changes relative to their respective control individuals, using the likelihood ratio test of the interaction effect between disease status (patients versus heathy individuals) and population (African Americans versus Chuvash people). We detected 730 genes exhibiting different expression changes between SCA and CE at 5% FDR, among which we further identified 453 genes that were among the 1226 differentially expressed genes in SCA reticulocytes and that showed no evidence of similarly altered expression in CE as defined by nominal P-value < 0.05. Of these 453 genes with “SCA-specific” expression changes, 300 had increased expression and 153 had decreased expression in SCA reticulocytes (STables 1 and 2). Figure 3B shows the top 20 most significant genes whose expression changes differed between SCA and CE.

The magnitude of expression changes that were shared between SCA and CE reticulocytes was in general greater in SCA (Fig. 3C), likely reflecting a robust hypoxic response to severe anemia in SCA. Genes with shared expression changes between SCA and CE had no enriched GO or KEGG at 5% FDR. Genes with SCA-specific increased expression are enriched in GO “ubiquitin-dependent protein catabolic process”, “protein polyubiquitination”, and “protein ubiquitination”. Genes with SCA-specific decreased expression are enriched in GO “cytoplasmic translation” and “translation” and KEGG "ribosome" and "coronavirus disease—COVID-19" (STable 5, Fig. 3D).

### Erythroid expression variation that correlates with hemoglobin concentration and hematocrit in SCA

PBMCs are easily obtainable peripheral blood cells normally comprised of lymphocytes, monocytes, dendritic cells^22^ and a small amount of erythroid progenitors^23,24^. PBMCs from SCA contain elevated amounts of erythroid progenitors due to accentuated erythropoiesis^9,17^. We correlated erythroid expression variation with clinical variation employing deconvolution analysis on PBMC expression data. Deconvolution analysis may use cell/tissue-specific marker genes to dissect gene expression variation in samples with cell/tissue heterogeneity^19^. The study included 151 SCA patients 19–72 years old under steady state (mean ± SD age 37 ± 12 years; 53% female; mean ± SD hemoglobin concentration 86 ± 14 g/L). For the 1226 genes differentially expressed in SCA reticulocytes, we first assessed their PBMC expression correlation with a 16-gene signature of erythroid progenitors^20^ in the 151 SCA patients. We identified 673 genes whose PBMC expression correlated with the erythroid gene signature, i.e., genes suitable for deconvolution analysis.

**Figure 4:**
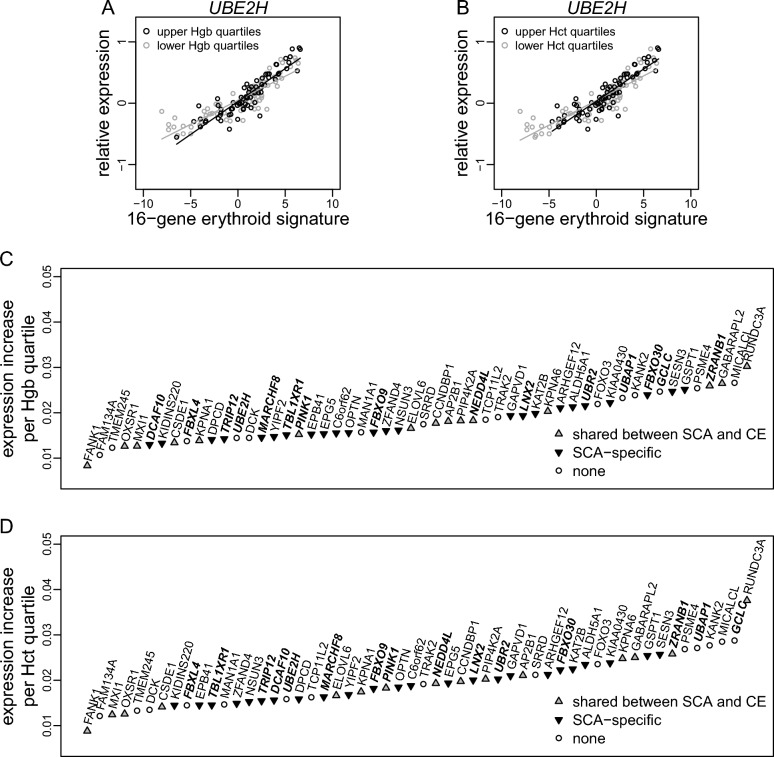
Correlation of erythroid expression of 54 genes with hemoglobin concentration (Hgb) and hematocrit (Hct). (**A,B**) Illustration for the *UBE2H* gene as an example, where the slope of PBMC expression of *UBE2H* versus the 16-gene erythroid signature was greater in patients with higher levels of (**A**) Hgb and (**B**) Hct. The significant erythroid signature by Hgb or Hct interaction effect (see “Methods”) indicates elevated erythroid expression of *UBE2H* with higher Hgb or Hct. (**C,D**) Increase in erythroid expression per quartile increase in (**C**) Hgb and (**D**) Hct. Gene symbols in bold text refer to genes annotated by GO keyword “protein ubiquitination”, “protein polyubiquitination”, and “ubiquitin-dependent protein catabolic process".

We correlated erythroid expression of the 673 genes in PBMCs with quartiles of hemoglobin concentration or hematocrit, adjusting for hemolysis defined as percent reticulocytes (“Methods”). When hemolysis is accounted for, more abundant hemoglobin or higher hematocrit signify more successful terminal differentiation and thus serve as proxies of more efficient erythropoiesis. The analysis was stratified by hydroxyurea treatment and restricted to 131 patients without blood transfusion. We detected 54 genes whose increased erythroid expression consistently correlated with higher hemoglobin concentration and higher hematocrit at 5% FDR (STable 6, Fig. 4). The expression of these genes was all increased in SCA reticulocytes relative to healthy individuals. Of the 673 genes subjected to deconvolution analysis, the 54 genes correlated with hemoglobin concentration and hematocrit are enriched with SCA-specific, increased expression (OR = 2.9, Fisher’s exact test P-value = 0.00063). The 54 genes are also enriched with GO annotations with key word “protein ubiquitination” (*DCAF10, FBXO30, FBXO9, GCLC, LNX2, NEDD4L, PINK1, UBR2*), “protein polyubiquitination” (*MARCHF8, NEDD4L, TRIP12, UBE2H*), and "ubiquitin-dependent protein catabolic process" (*FBXL4, FBXO9, GCLC, NEDD4L, PINK1, TBL1XR1, TRIP12, UBAP1, UBE2H, UBR2, ZRANB1*) (OR = 4.2, P-value = 7.5 × 10^–5^).

### An erythroid eQTL associating with severe pain episodes in SCA

eQTL are genetic polymorphisms associated with gene expression variation. We mapped erythroid eQTL on the same PBMC expression data from the 151 SCA patients for the 673 genes suitable for deconvolution analysis as in the previous section (“Methods”). At 5% FDR, we detected 180 associations between 180 local single nucleotide polymorphisms (SNPs), i.e., SNPs located less than 100 Kb away from gene ends, and erythroid expression of 18 genes. The Genotype-Tissue Expression (GTEx) project generates categories of eQTL across diverse tissues^25^ which currently do not cover erythroblasts. Because of substantial sharing of eQTL across tissues^26^, a comparison of erythroid eQTL with GTEx eQTL from diverse tissues may assure the validity of the deconvolution analysis. Of the 180 gene-SNP associations, 117 (65%) overlapped with eQTL associations from GTEx analysis version 8 for at least one tissue, representing a tenfold enrichment and supporting our erythroid eQTL results.

We tested genetic association of the 180 SNPs with severe pain episodes, defined as having three or more pain episodes that required Emergency Room visit or hospitalization in the past 12 months^27^, in 774 SCA patients from the Walk-PHaSST^28^, PUSH^29^, and UIC cohorts (STable 7). The genetic association results are present in STable 8. The most significant association, between an eQTL of *LNX2* and severe pain episodes, reached Bonferroni-corrected P < 0.05 (STable 8). *LNX2* encodes ligand of Numb-protein X 2 and is annotated by GO “protein ubiquitination”. Expression of *LNX2* was specifically increased in SCA reticulocytes (STable 1). Erythroid expression of *LNX2* correlated with higher hemoglobin concentration and hematocrit (STable 6). The eQTL, rs7992289, is 83,620 bp down stream of *LNX2*. The G allele of the eQTL (frequency = 0.29; imputation quality r^2^ = 0.97) associated with greater expression level of *LNX2* (β = 0.043, 95% CI 0.024–0.062, P = 2.4 × 10^–5^) and with severe pain episodes (OR = 1.7, 95 CI 1.3–2.2, P = 3.9 × 10^–5^) (Table 1).

**Table 1:** Association of the G allele of rs7992289 with erythroid gene expression of *LNX2* using linear model and with severe pain episodes ≥ 3 using binary logistic model.

Trait	Walk-PHaSST			Push			UIC			Combined		
	OR (95% CI)	P-value	N	OR (95% CI)	P-value	N	β or OR (95% CI)	P-value	N	OR (95% CI)	P-value	N
Erythroid Expression of *LNX2*							0.043 (0.024–0.062)	2.4 × 10^–5^	151			
Severe pain episodes ≥ 3	1.4 (0.98–1.9)	0.062	399	2.5 (1.5–4.2)	0.00048	207	1.9 (1.1–3.4)	0.022	168	1.7 (1.3–2.2)	3.9 × 10^–5^	774

*OR* odds ratio.

## Discussion

Analysis of the reticulocyte transcriptome in SCA suggests heightened differentiation in early erythroblasts that diminishes toward transition to orthochromatic stage. Comparison with reticulocyte transcriptome in CE reveals SCA-specific upregulation of genes involved in ubiquitin-dependent protein degradation and protein ubiquitination. Deconvolution analysis in SCA PBMCs identified 54 genes whose erythroid expression correlated with erythropoiesis efficiency, which are enriched with SCA-specific upregulation and with functional annotation of ubiquitin-dependent protein degradation and protein ubiquitination. An erythroid eQTL of one such gene, *LNX2*, was found to associate with severe pain episodes in 774 SCA patients. Our findings raise a hypothesis that increased ubiquitin-dependent protein degradation and protein ubiquitination in late erythroid differentiation may link improved erythroblast survival with altered presentation of surface proteins in SCA erythroid cells that favors adherence to the microvasculature, a feature of SCA pain crisis.

A marked number of genes whose expressions are normally decreased in transition from polychromatic to orthochromatic stage had increased expression in SCA reticulocytes (Fig. 2C), implying distinct cellular conditions in the latest stages of SCA erythroid differentiation. These include genes that normally showed transiently increased expression in the polychromatic stage and that are enriched in GO “ubiquitin-dependent protein catabolic process” (Fig. 2C). Transient transcriptional activation of genes encoding ubiquitin conjugation enzymes has been observed in normal erythropoiesis^30^, potentially to eliminate misfolded proteins at the peak of hemoglobin synthesis^31^. Polymerization of HbS can occur in the hemoglobin synthesis phase and induce apoptosis in erythroblasts derived from SCA patients^9,32^. Apoptosis of erythroblasts in SCA was associated with cytoplasmic sequestration of HSP70 similary to β-thalassemia^33^, which can be rescued by induction of HbF synthesis to interfere with HbS polymerization^9^. In β-thalassemia, the ubiquitin–proteasome system plays a critical role in proteolytic control of globin chain balance^34^. Our study identified 54 genes whose erythroid expression correlated with more effective erythropoiesis as reflected by higher hemoglobin concentration and hematocrit. The 54 genes are enriched with annotation keyword “ubiquitin-dependent protein catabolic process”, “protein polyubiquitination”, and “protein ubiquitination”, suggesting that upregulation of these processes may alleviate oxidative damges^35^ resulted from HbS polymerization in SCA erythroblasts.

The ubiquitin system contributes to membrane remodeling, in addition to protein quality control and autophagy which eliminates mitochondria and ribosomes^36^. Ubiquitination of plasma membrane aquaporin-1 is required for its endocytosis, sorting, and secretion^37^. In the presence of bulk protein damage, the ubiquitin–proteasome system can be overloaded and impact the turnover of plasma membrane proteins^38^. In β-thalassemia, membrane assembly of spectrin, band 3, and band 4.1 in erythroblasts is disrupted^39^. In SCA, the adhesive markers glycoprotein IV^40^ and α4β1 integrin receptor^15^ are uniquely displayed on the reticulocyte surface^4^. Hydroxyurea treatment, which reduces HbS polymerization by elevating HbF level, decreases these markers on the surface of reticulocytes and erythroblasts in children with SCA^41^ and decreases the adhesion of sickle erythrocytes to thrombospondin and laminin in vitro^42^. We found that the G allele of SNP rs7992289 associates with increased erythroid expression of *LNX2* and increased risk for severe pain episodes in SCA patients. The *LNX2* gene encodes an E3 ubiquitin ligase that was shown to promote CD8α ubiquitination, endocytosis, and degradation in lysosomes^43^. The results imply that the *LNX2* gene may have contrary impacts in SCA erythroblast survival and reticulocyte maturation. LNX2 was also shown to modulate the ubiquitination status of the neuronal glycine transport GlyT2^44^, the gene expression level of which in PBMCs was found to positively correlate with pain frequency in our patients^45^. Future studies are needed to confirm these associations and elucidate LNX2 substrates in SCA erythroblasts.

There are several limitations of this study. First, it compared relatively small number of hemoglobin SS patients and hemoglobin AA controls using reticulocyte RNA-Seq. The detection of 10.4% of the analyzed genes as differentially expressed genes with this small sample size, however, suggests that large transcriptional differences accumulate during SCA erythropoiesis. The transcriptional differences can be attributed to distinct selection pressure in SCA bone marrow^9^, disturbed progression of erythroblast maturation, and varied rates of RNA degradation, in addition to transcriptional changes at aligned differentiating stages. As such, the second limitation of this study is the lack of biological resolution with the current experimental assays. Our reticulocyte purification protocol inclusively collects reticulocytes in peripheral blood (“Methods”). A finer delineation of the heterogeneous reticulocytes may be accomplished by single cell RNA-Seq. A more resource-demanding approach using in vitro derived erythroblasts may further supplement the study. Third, the comparison between SCA and CE was confounded with both ethnicity and experimental batch effects. Our study design ameliorates such confounding in that patients were paired with control individuals matched for ethnicity, age, gender, and experimental batch. Despite of these limitations, our results suggest that a deeper understanding of transcriptome dysregulation of erythroid cells in SCA has potential to shed novel insights on SCA pathogenesis.

## Methods

### Ethics approval and consent to participate

Institutional Review Board (IRB) of UIC approved the study “Genomic and Biomarker Studies in Sickle Cell Disease” for the UIC cohort (protocol # 2010-1125). IRBs of Albert Einstein College of Medicine, Columbia University, Children's Hospital Oakland, Children's Hospital Pittsburgh, Hammersmith Hospital London, Howard University, Johns Hopkins University, NHLBI, University of Colorado, and UIC approved the study “Treatment of Pulmonary Hypertension and Sickle Cell Disease with Sildenafil Therapy Walk-PHaSST” for Walk-PHaSST (protocol # 2007-0936). IRBs of Children’s National Medical Center, Howard University Hospital, University of Michigan Ann Arbor, and Pulmonary and Vascular Medicine Branch of the NHLBI approved the study “Pulmonary Hypertension and the Hypoxic Response in SCD” for PUSH (protocol # 2011-1071). All subjects provided written informed consent to participate. This study was designed and carried out in accordance with the Declaration of Helsinki.

### Patients and patient cohorts

Five SCA patients and 5 African-American control individuals from UIC were studied for differential gene expression in SCA reticulocytes. Seventeen CE patients and 13 control individuals from Chuvashia, Russia were studied for differential gene expression in CE reticulocytes. To assess clinical correlation with erythroid gene expression we studied 151 SCA patients from UIC whose PBMC expressions were profiled by arrays previously^46^. To assess genetic association with disease manifestations, we studied 774 SCA patients: 399 patients from Walk-PHaSST, a screening study that enrolled patients ≥ 12 years old at 10 centers from 2008–2009^28^, 207 patients from PUSH, which enrolled patients 3–20 years old at four centers from 2006 to 2010^29,47^, and 168 UIC patients including the 151 selected for PBMC expression analysis. All patients were in their usual state of health.

### Reticulocyte RNA-Seq data and analysis

Whole blood (8–16 mL) was collected using ACD as an anticoagulatent. Plasma and platelets were isolated by gentle centrifugation at 400 g. Blood was further layered on ficoll-histopaque (density 1.077 g/mL) for separation of mononuclear cells and lymphocytes. The lower layer of packed erythrocytes were transferred to a clean tube and preferentially lysed with 4 volumes of 0.144 M ammonium chloride, 1 mM ammonium bicarbonate solution. Granulocytes were separated by centrifugation and the supernatant containing reticulocyte RNA was precipitated by acid titration to pH 5.1. Precipitated RNA was preserved in Trizol and stored at − 80 °C^48^ Library construction was performed using the Illumina TruSeq Stranded Total RNA Sample Preparation Kit with Ribo-Zero Human/Mouse/Rat. Strand-specific libraries were constructed for 100 or 125 bp paired-end sequencing to 30–45 million read pairs per sample using Illumina HiSeq 2500 or 4000 platform. RNA extraction, library construction, and sequencing were performed at the University of Chicago for SCA and control samples in one batch and at the University of Utah for CE and control samples in three batches. Sequencing data were mapped to human reference genome version GRCh37 annotated by Gencode version 24, using STAR^49^. The effect of SCA or CE on autosomal gene expression was assessed by the likelihood ratio test based on the negative binomial distribution as implemented in DESeq2^50^. Batch effects were adjusted in the analysis of CE and controls. Genome-wide comparison of SCA with CE was assessed by the likelihood ratio test of the interaction effect between disease status (patients vs. heathy individuals) and population (African Americans versus Chuvash people), controlling for batch effects. NIH DAVID^51^ was used for gene enrichment in GO^52^ and KEGG^53^.

### Deconvolution of erythroid gene expression using PBMC data

Messenger RNA was purified from PBMCs from 151 UIC SCA patients and profiled on Affymetrix Gene 2.0 ST Array. Probes with unique perfect alignment to human genome assembly GRCh37 were selected. Probes that interrogate transcripts from multiple genes were removed, as well those that contain SNPs with ≥ 1% minor allele frequency (MAF) in dbSNP release 149 to avoid signal noise. Probe intensities were log2 transformed, background corrected, and quantile normalized. The corresponding probe mean across samples was then subtracted from the probe intensity. Gene expression level was summarized as average intensity across probes according to Gencode version 24. Experimental batch effects were adjusted using an empirical Bayes method^54^.

We assessed correlation of erythroid gene expression in PBMCs with hemoglobin concentration using the model:

${y}_{ij}={u}_{i}+{x}_{j}^{ery}\cdot {\beta }_{i}^{ery}+{x}_{j}^{hgb}\cdot {\beta }_{i}^{hgb}+{{{x}_{j}^{hemo}\cdot \beta }_{i}^{hemo}+x}_{j}^{int\_hgb}\cdot {\beta }_{i}^{int\_hgb}{+{ x}_{j}^{int\_hemo}\cdot {\beta }_{i}^{int\_hemo}+\varepsilon }_{ij}$. Here ${y}_{ij}$ denotes expression of gene $i$ for patient $j$. ${u}_{i}$ denotes mean expression of gene $i.$ ${x}_{j}^{ery}$, ${x}_{j}^{hgb}$, ${x}_{j}^{hemo}$, ${x}_{j}^{int\_hgb}$, ${x}_{j}^{int\_hemo}$ denote the﻿ number of erythroid progenitors, hemoglobin concentration in quartiles, level of hemolysis reflected by square root transformed percent reticulocytes, erythroid progenitor by hemoglobin concentration interaction, and erythroid progenitor by hemolysis interaction, respectively for patient $j$. $\beta$ s denotes the corresponding coefficients for gene $i$. ${\varepsilon }_{ij}$ denotes residual error. The P-value of the erythroid progenitor by hemoglobin concentration interaction term, representing erythroid gene expression correlation with hemoglobin concentration, was estimated by the F test. Correlation of erythroid gene expression with hematocrit was similarly assessed. The number of erythroid progenitors was approximated as the first principal component of PBMC gene expression of 16 marker genes^20^.

For erythroid eQTL mapping, PBMC gene expression levels were analyzed using the model:

${y}_{ij}={u}_{i}+{x}_{j}^{ery}\cdot {\beta }_{i}^{ery}+{x}_{j}^{dose}\cdot {\beta }_{i}^{dose}+{x}_{j}^{int}\cdot {\beta }_{i}^{int}+{{x}_{j}^{sv}\cdot {\beta }_{i}^{sv}+\varepsilon }_{ij}$. Here ${x}_{j}^{dose}$ denotes SNP allele dosage, ${x}_{j}^{int}$ denotes erythroid progenitor by allele dosage interaction, and ${x}_{j}^{sv}$ denotes surrogate variables^55^ for patient $j$. The P-value of the erythroid progenitor by allele dosage interaction term, representing erythroid gene expression correlation with allele dosage, was estimated by the F test.

### Genetic association between erythroid eQTL and clinical manifestations

Genomic DNA isolated from PBMCs was genotyped using Illumina Human 610-Quad SNP array for Walk-PHaSST and PUSH patients and Affymetrix Axiom Pan African array for UIC patients. Samples having a genotype rate < 95% were removed. SNPs deviating from Hardy Weinberg equilibrium (P-value < 0.0001) or with MAF < 0.01 were removed. Population outliers were identified by principal components analysis using PLINK 2.0^56^ and removed from analysis. Kinship coefficients were estimated using KING-robust^57^. First to third-degree relatives as well as duplicates were randomly thinned to one individual per family. Genotypes were phased using SHAPIT2^58^ and imputed to 1000 genomes project phase 3 data with African reference panels using IMPUTE2^57^. To provide an empirical estimation of the imputation quality, chromosome 22 from the UIC patients was re-imputed with 5% of array-genotyped SNPs randomly masked, using the same parameters for whole genome imputation. The median (interquartile range) of concordance rate between imputation and array-genotyping is 0.97 (0.95–0.99).

SNPs with MAF > 0.1 and imputation r^2^ > 0.9 were further tested for erythroid expression association in UIC patients. Erythroid eQTL were assessed for association with severe pain episodes. We examined binary outcome of having 3 or more pain episodes requiring emergency room visits or hospital admission over 12 months prior to a baseline visit^27^, using logistic regression with covariates of age, gender, α-thalassemia as copies of deletion, and population stratification. P-value was estimated by Wald test. Meta-analysis used inverse variance based method^58^.

R version 4.2.1^59^ was used in regression analyses. In all of the analyses, multiple comparison was controlled by Benjamini–Hochberg procedure^60^ and significance was defined as adjusted P-value < 0.05, unless specified otherwise.

### Supplementary Information

Supplementary Figure 1.

Supplementary Tables.

## Data Availability

The datasets generated during the current study are available in the GEO repository with accession number GSE232221: https://www.ncbi.nlm.nih.gov/geo/query/acc.cgi?acc=GSE232221.
