# Publisher Correction: Gene expression changes in sickle cell reticulocytes and their clinical associations

**DOI:** 10.1038/s41598-023-42020-5

**Published:** 2023-09-14

**Authors:** Xu Zhang, Jihyun Song, Binal N. Shah, Jin Han, Taif Hassan, Galina Miasniakova, Adelina Sergueeva, Sergei Nekhai, Roberto F. Machado, Mark T. Gladwin, Santosh L. Saraf, Josef T. Prchal, Victor R. Gordeuk

**Affiliations:** 1 Department of Medicine, University of Illinois at Chicago, Chicago, IL USA; 2 Department of Medicine, University of Utah, Salt Lake City, UT USA; 3 College of Pharmacy, University of Illinois at Chicago, Chicago, IL USA; 4 Chuvash Republic Clinical Hospital 2, Cheboksary, Russia; 5 Cheboksary Children’s Hospital, Cheboksary, Russia; 6 Center for Sickle Cell Disease, Howard University, Washington, DC USA; 7 Division of Pulmonary, Critical Care, Sleep, and Occupational Medicine, Department of Medicine, Indiana University, Indianapolis, IN USA; 8 Division of Pulmonary, Allergy, and Critical Care Medicine, Vascular Medicine Institute, University of Pittsburgh, Pittsburgh, PA USA

Correction to: *Scientific Reports* 10.1038/s41598-023-40039-2, published online 08 August 2023

The original version of this Article contained errors in Figure 3. The numbers in the colour bar of Figure 3B ‘-3, -2, -1, 0, 1, 2, 3’ did not align correctly with the gradients of the colour bar, the two characters "va" within the word "Control-Chuvash" in the legends of grouping annotation of Figure 3A, B overlapped and "Coronavirus disease - Covid-19" in the y-axis labelling in Figure 3D did not align correctly. The original Figure 3 and accompanying legend appear below.

**Figure 3:**
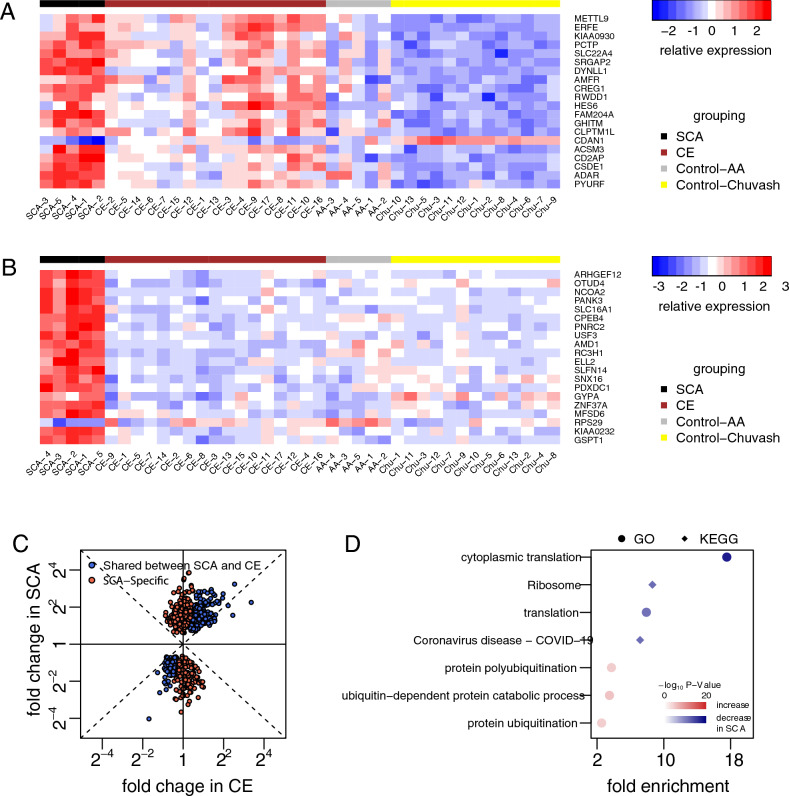
SCA-specific expression changes. (**A,B**) Hierarchical clustering of individuals within groups, using Euclidean distance of gene expression levels of (**A**) the top 20 most significant differential genes in CE whose expression changes were shared in SCA and (**B**) the top 20 most significant genes whose expression changes differed between SCA and CE. Individuals are grouped as SCA, CE, African American controls (Control-AA), and Chuvash controls (Control-Chuvash). (**C**) Scatter plot of fold change in CE (x-axis) and SCA (y-axis). (**D**) GO biological processes and KEGG pathways enriched with SCA-specific differential genes.

The original Article has been corrected.

