# Supplementary material for: Gene expression changes in sickle cell reticulocytes and their clinical associations
Source: Sci Rep. 2023 Aug 8;13:12864. doi: 10.1038/s41598-023-40039-2 (PMC10409856; doi:10.1038/s41598-023-40039-2)
Supplement: Supplementary file 1 — Supplementary Figure 1. [file 41598_2023_40039_MOESM1_ESM.doc]

**Supplemental Figure 1**. Principal components analysis of transcriptomes from five SCA patients and five African American control individuals.
